# Determination of new generation amide insecticide residues in complex matrix agricultural food by ultrahigh performance liquid chromatography tandem mass spectrometry

**DOI:** 10.1038/s41598-021-02645-w

**Published:** 2021-12-01

**Authors:** Tao Lin, Xinglian Chen, Li Wang, Haixian Fang, Maoxuan Li, Yangang Li, Hongcheng Liu

**Affiliations:** 1 Quality Standards and Testing Technology Research Institute, Yunnan Academy of Agricultural Science, Beijing Road 2238 Number, Kunming, 650205 People’s Republic of China; 2 Laboratory of Quality & Safety Risk Assessment for Agro-Products (Kunming), Ministry of Agriculture and Rural Affairs, Kunming, 650205 People’s Republic of China

**Keywords:** Environmental sciences, Chemistry

## Abstract

Eight new generation amide insecticide residues analysis by multiwalled carbon nanotubes (MWCNs) cleanup, combined with QuEChERS and ultrahigh performance liquid chromatography tandem mass spectrometry has been developed and successfully applied in complex matrix such as orange, celery, onion, litchi, mango, shallot, chives, avocado, garlic. The matric effect of MWCNs is optimized and compared with ordinary cleanup materials. The results show that the performance of MWCNs is fine and effectively reduce matrix interference. Through chemical structure skeletons analyzed, chlorantraniliprole, bromoantraniliprole, and cyantraniliprole can cause same product ions of m/z 286.0 or 177.1 in the ESI^+^ mode, then tetrachlorantraniliprole and cyclaniliprole can produce collective ions of m/z 146.9 in the ESI^−^ mode. The coefficients (*R*^2^) were greater than 0.9990, the limit of quantification ranges from 0.03 to 0.80 μg/kg, the recovery rate ranges from 71.2 to 120%, and the relative standard deviation (RSD) ranges from 3.8 to 9.4%. The method is fast, simple, sensitive, and suitable for the rapid determination of amide pesticides in complex matrix agricultural food.

## Introduction

New generate amide insecticides are a novel class of pesticides, because of their high efficiency and low toxicity. The early products include chlorantraniliprole, cyantraniliprole, flonicamidand and flubendiamide^1^, whereas cyclaniliprole and tetrachlorantraniliprole, bromoantraniliprole are the more recent members. They can activate insect ryanodine receptors, which play a critical role in muscle function^2^. They can be used to against a variety of insects, such as *Cydia pomonella* in apple, and *Plutella xylostella* (Linnaeus) in cabbage and pakchoi etc^3^.

New amide insecticides are currently generally considered low-toxic pesticides. However, according to the test results of flubendiamide's environmental behavior and ecological toxicology, it has shown that it has unacceptable risks to invertebrates (*Daphnia magna*) and may have great risk to the aquatic ecological environment. Therefore, the Ministry of Agriculture and Rural Affairs of China banned the use of flubendiamide in rice in 2018. According to China’s National Food Safety Standard-Maximum residue limits for pesticides in food (GB 2763-2021), the ADI (Acceptable daily intake body weight) for tolfenpyrad is 0.006, indicating that it has a certain degree of toxicity. Due to the protection of foreign compound patents, pesticides such as chlorantraniliprole, bromoantraniliprole and cyclaniliprole have not been legally registered in European Union (the Annex of Regulation (EC) No 396/2005), but monitoring these insecticides are particularly important and urgent for assessing the food safety risk or for setting MRLs in plants.

There have been a number of analytical methods for the diamide residue determination, Tian reported simultaneous determination of five diamide insecticides in mushroom^3^, in food matrices^4,5^, chlorantraniliprole and flubendiamide in vegetable^6^, chlorantraniliprole and cyantraniliprole in food^7^, and in soil^8^. The residue analysis of bromoantraniliprole is rarely reported to date. Some challenges are also identified for the analytic method for pesticide residue. The most complicated one is the influence of complex sample matrix on the extracted amount of target analyte. The effects of complex matrix generally will enhance or inhibit mass signal to cause the result error over the method, the complex matrix includes green onions, leeks, citrus.

At present, the QuEChERS method is widely used in the rapid determination of pesticides in agricultural products because of its rapidness, simplicity, low cost, high efficiency and environmental friendliness^9–11^. In conventional QuEChERS clean-up steps with graphitized carbon black (GCB)^11^, PSA^9^, C18^10^ as adsorbent have also been reported. Multiwalled carbon nanotubes (MWCNTs) have also been widely used in the purification process of complex agricultural products in recent years, and they have a good effect on adsorbing pigments and reducing matrix effects^12^.

This study is to develop a method modified QuEChERS for simultaneous determination of eight new generate amide insecticides including chlorantraniliprole, bromoantraniliprole, flonicamid, cyantraniliprole, tolfenpyrad, flubendiamide, tetrachlorantraniliprole and cyclaniliprole in fruits and vegetables by ultrahigh performance liquid chromatography tandem mass spectrometry (UPLC-MS/MS). The complex matrix of green onions, celery, leeks, citrus, lychees, avocado, are extracted and cleaned by MWCNTs.This method could be very practical for fast screening the new generate amide insecticides in vegetable and fruit to ensure food safety.

## Experimental procedures

### Chemicals and materials

Standards of chlorantraniliprole, bromoantraniliprole, flubendiamide, tetrachlorantraniliprole, cyclaniliprole, flonicamid, cyantraniliprole and tolfenpyrad were bought from Tianjin Alta Scientific Ltd. (Tianjin, China), and all those compounds were dissolved in methanol to 100 µg/mL. Methanol and acetonitrile of HPLC grade were obtained from Merck KGaA. (Darmstadt, Germany). Highly purified water was prepared by a Milli-Q water purification system (Bedford, MA). Ammonium formate (≥ 99.995%) was purchased from MilliporeSigma Company (St Louis, MO). Analytical reagent grade including anhydrous sodium chloride (NaCl) and magnesium sulfate (MgSO_4_) were obtained from Sinopharm Chemical Reagent (Beijing, China). MWCNTs (10–30 µm length, 10–20 nm diameter) were provided by Nanjing XFNANO Materials Tech Co., Ltd. (Nanjing, China). PSA with diameter of 50 μm were bought from Dikma Technologies Inc. (Beijing, China).

### Instruments

Sample analyses were performed on An AB Sciex API4000 mass spectrometer (MS/MS) (Framingham, MS) coupled to a 1290 Ι Infinity UHPLC (Agilent technology, USA). Waters ACQUITY UPLC BEH C18 column (2.1 × 50 mm, 1.7 μm) was obtained good resolution. Solvents A (1 mM ammonium formate in ultrapure water with 0.1% formic acid) and B (methanol) were used at a flow rate of 0. 2 mL/min with the following gradient: 55% B → 95% B (3.0 min) → 95% B (4.5 min) → 55% B (4.7 min) → 55% B (6.0 min). The injection volume was 1 µL.

The electrospray ionization (ESI) source was operated in both positive (ESI^+^) and negative (ESI^−^) mode for simultaneously forming [analyte + H]^+^ and [analyte − H]^−^ ions. Analyte ion transitions used for qualification and quantitation were monitored by multiple reaction monitoring (MRM) mode. Ion source conditions were used as follows: ionspray voltage, 5500 V (ESI^+^)/− 4500 V (ESI^−^); heating gas temperature, 550 °C; curtain gas flow rate, 20 L h^−1^; nebulizing gas flow rate, 55 L h^−1^; heating gas flow rate, 55 L h^−1^. The identification of proper ion transitions (precursor ion > product ion) of each amide pesticide and the optimization of a number of MS/MS parameters including declustering potential (DP) and collision energy (CE) were performed with a syringe pump providing a constant flow of the standard solution (0.1 μg mL^−1^) of amide pesticide to the MS/MS at a flow rate of 10 μL min^−1^. The parameters for the detection of the eight amide pesticides are shown in Table 1.

**Table 1:** UHPLC–MS/MS parameters for detection of the eight amide pesticides.

Compound	Ionization mode	Precursor ion (*m/z*)	Product ion (*m/z*)	DP (V)	CE (V)
Chlorantraniliprole	ESI^+^	484.1	453.0^a^/286.0	58	24/29
Bromoantraniliprole	ESI^+^	527.9	286.0^a^/177.1	60	24/68
Flonicamid	ESI^+^	230.0	98.0^a^/146.0	73	53/43
Cyantraniliprole	ESI^+^	475.0	286.0^a^/177.1	63	19/28
Tolfenpyrad	ESI^+^	384.0	197.1^a^/154.1	60	36/57
Flubendiamide	ESI^−^	681.0	253.8^a^/272.0	− 90	− 37/− 24
Tetrachlorantraniliprole	ESI^−^	537.9	203.9^a^/146.9	− 62	− 16/− 47
Cyclaniliprole	ESI^−^	602.0	257.9^a^/146.9	− 80	− 21/− 47

^a^Quantitative ion.

### Sample preparation

All vegetable and fruit samples were collected from supermarkets or farmer’s markets with the permission of local management personnel, and all the supermarkets or farmer’s markets are legally registered locally. The experiment was performed in accordance with the regulations (NY/T 789-2004) established by the Ministry of Agriculture and Rural Affairs of the People’s Republic of China. A thoroughly homogenized vegetable or fruit sample (10 g) was weighted into a 50 mL Teflon centrifuge tube, then 10 mL acetonitrile was added. The tube was shaken vigorously for 2 min with vortex mixer to ensure entirely solvent extraction. Anhydrous NaCl (1 g) and anhydrous MgSO_4_ (4 g) were added into the solution and the shaking step was repeated for 1 min. After centrifugation (5000 rpm, 3 min), 2 mL supernatant solution was transferred into a 10 mL Teflon centrifuge tube containing 20 mg MWCNTs and 300 mg MgSO_4_. Then the mixture was shaken vigorously for 1 min and centrifuged for 3 min at 5000 rpm. Finally the extraction layer was filtered by 0.22 μm filter membrane and determined by LC–MS/MS.

### Method validation

The following parameters were validated according to SANTE/11813/2017^13^, the quantification limits, linear ranges and correlation coefficients of each amide pesticide in different matrices were determined.

Recovery and reproducibility experiments was tested for each matrix in six replicates each at three fortification levels (Limit of quantification, five times limit of quantification, ten times limit of quantification).

To evaluate matrix effect, nine representative samples were selected. Onions, shallots, leeks and garlic were selected as representative commodity with high irritating sulfide content, orange including high acidic and volatile oils content, lychee and mango containing high sugar content. Matrix effects (ME) were measured according to the equation^14^:

$$ME = \left( {{\text{slope of solvent standard}}/{\text{slope of matrix matched standard}} - {1}} \right) \times {1}00\%$$

when the result of *ME* < 0, it means matrix inhibitory, > 0 means matrix enhancement, *ME* is 0–20% meaning weak matrix effect, medium matrix effect with 20–50%, more than 50% meaning strong matrix effect.

## Results and discussion

### Mass spectrometry optimization

In the ESI^+^ and ESI^−^ modes, the precursor and product ions were scanned through pesticide standards (0.1 μL mL^−1^) respectively, and the mass spectrometry were optimized in Table 1.

**Figure 1:**
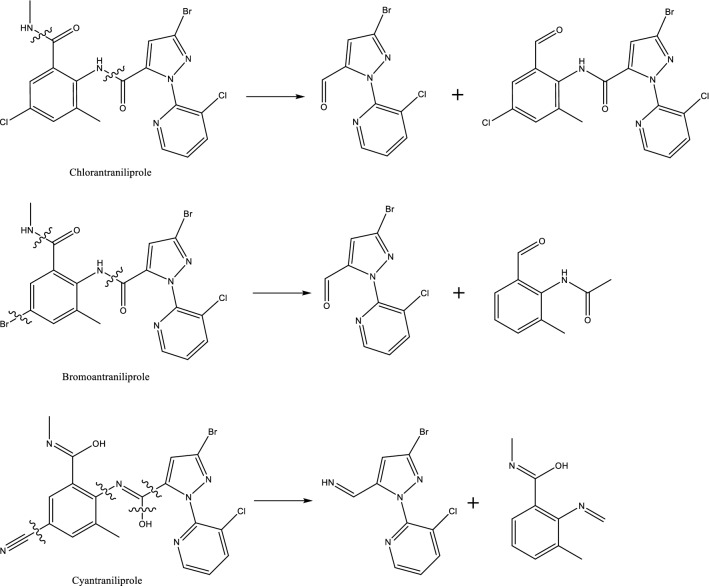
Three amide pesticides broken in positive mode.

The mass spectrum signals of chlorantraniliprole, bromoantraniliprole and cyantraniliprole in the ESI^+^ mode were better enhanced than those in the ESI^−^ mode due to their similar chemical skeletons. In the positive ion mode, three pesticides caused same product ions of m/z 286.0 or 177.1. The amide bonds in chlorantraniliprole and bromoantraniliprole were broken to produce m/z 286.0, then the breaking of amide bond and C–Br bond was formed m/z 177.1. The C=N bonds of cyantraniliprole were simultaneous broken to produce m/z 286.0 and 177.1 (Fig. 1).

**Figure 2:**
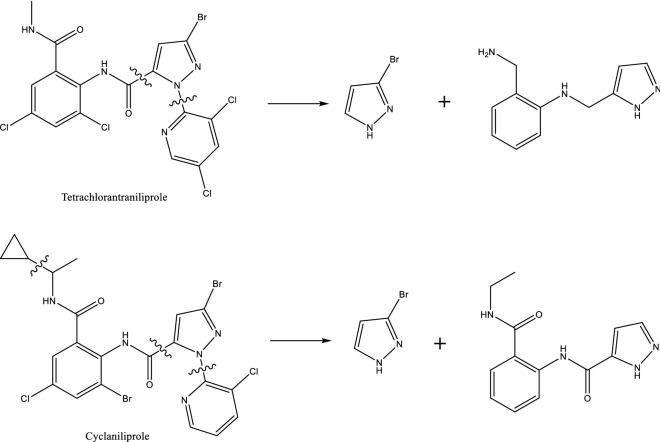
Tetrachlorantraniliprole and cyclaniliprole broken in negative mode.

In the negative mode, both tetrachlorantraniliprole and cyclaniliprole can produce same ions of m/z 146.9, which may be the 3-bromo-1H-pyrazole. The fragmentations of the secondary mass spectrometry were speculated as shown in Fig. 2.

**Figure 3:**
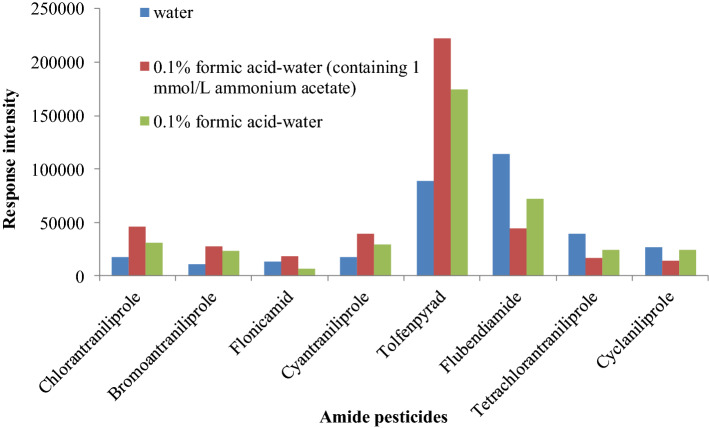
Response intensity of amide pesticides in different mobile phases.

**Figure 4:**
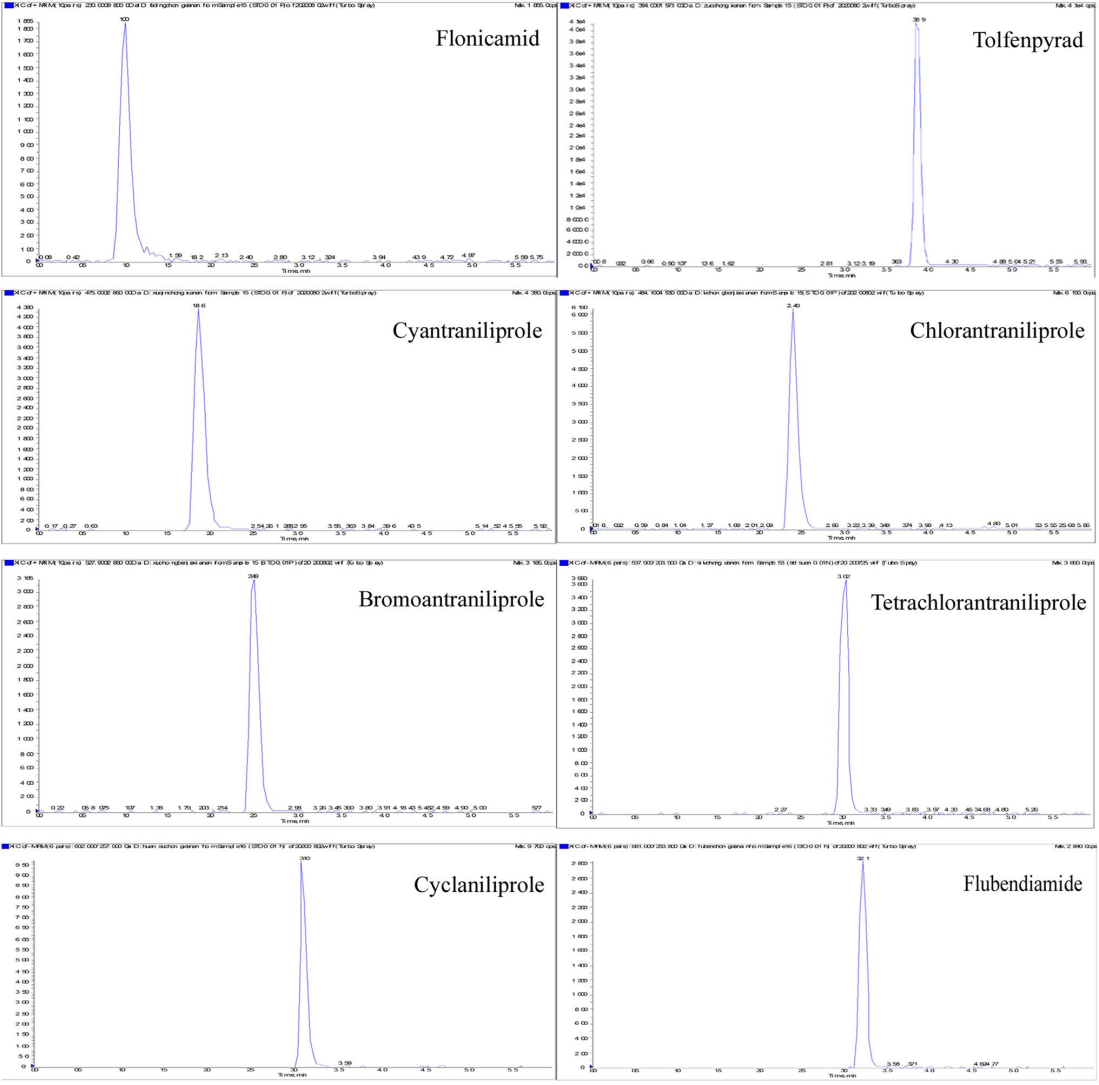
Chromatograms of amide pesticides.

In mass spectrometry analysis, the signal response was enhanced and the peak shape was improved when volatile salt and acid was added to the mobile phase^11,15,16^. Therefore, different mobile phase of 0.1% formic acid–water, 0.1% formic acid–water (containing 1 mmol L^−1^ ammonium acetate) and pure water were compared in the experiment (Fig. 3). Generally in the positive ion mode, the ionization efficiency was improved and the response intensity was enhanced when the acid and ammonium salt are added. Therefore, the response intensity of chlorantraniliprole, bromoantraniliprole, cyantraniliprole and tolfenpyrad were significantly improved when formic acid and ammonium acetate was added as the mobile phase. However, for flonicamid, the response was decreased in formic acid solution, but significantly increased by ammonium acetate. The same results exist in tetrachlorantraniliprole and cyclaniliprole. In the negative mode, the [M–H]^−^ ion was inhibited by excess hydrogen ion and ammonium ion result to decrease its response. The chromatograms are shown in Fig. 4.

### Matrix effects

**Figure 5:**
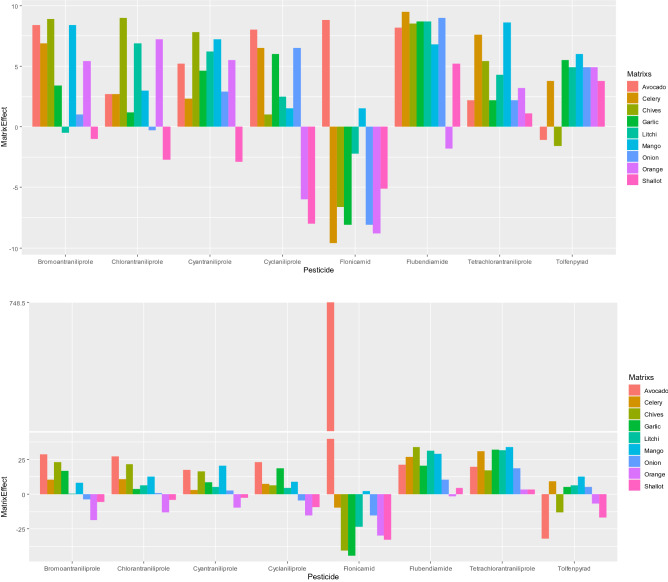
Matrix effect of amide pesticides under extraction conditions (**A**) cleaned up, (**B**) without cleanup.

As shown in Fig. 5, eight amide compounds have strong matrix effects, especially for the avocado with higher oil content, leeks and garlic with more sulfur compounds (matrix effect was: − 44.1 to 748.5%). However, with the addition of multiwalled carbon nanotubes, the matrix effect can be effectively suppressed, and better results have been obtained.

### Modified cleanup process

#### Optimization of the amount of the MWCNTs

It was found in the experiment that the amount of multiwalled carbon nanotubes can affect the recovery. To optimize this parameter, the experiment was performed by different amounts of MWCNTs (10, 20, 30 mg). With amount of multiwalled carbon nanotubes increasing, the recoveries slightly decreased. The recoveries of chlorantraniliprole, bromoantraniliprole and cyantraniliprole in onions were 53.3%, 45.9% and 54.5% respectively, and flonicamid in leek, cyantraniliprole in garlic were lower than 70% with 30 mg MWCNTs. Consequently, amount of MWCNTs was selected as 20 mg.

#### Comparison of purification material

**Figure 6:**
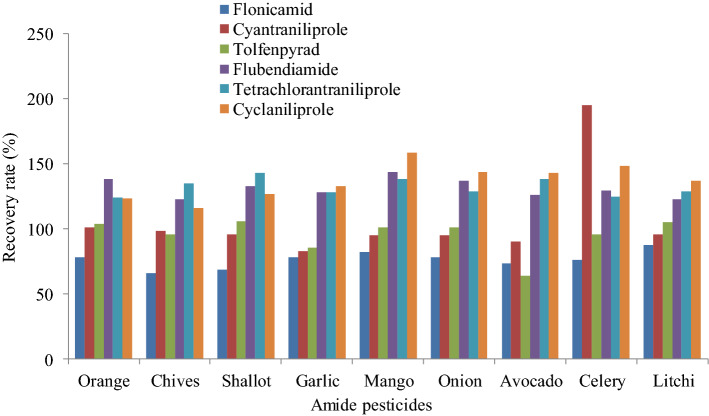
Different recovery of amide pesticides with PSA purification.

**Figure 7:**
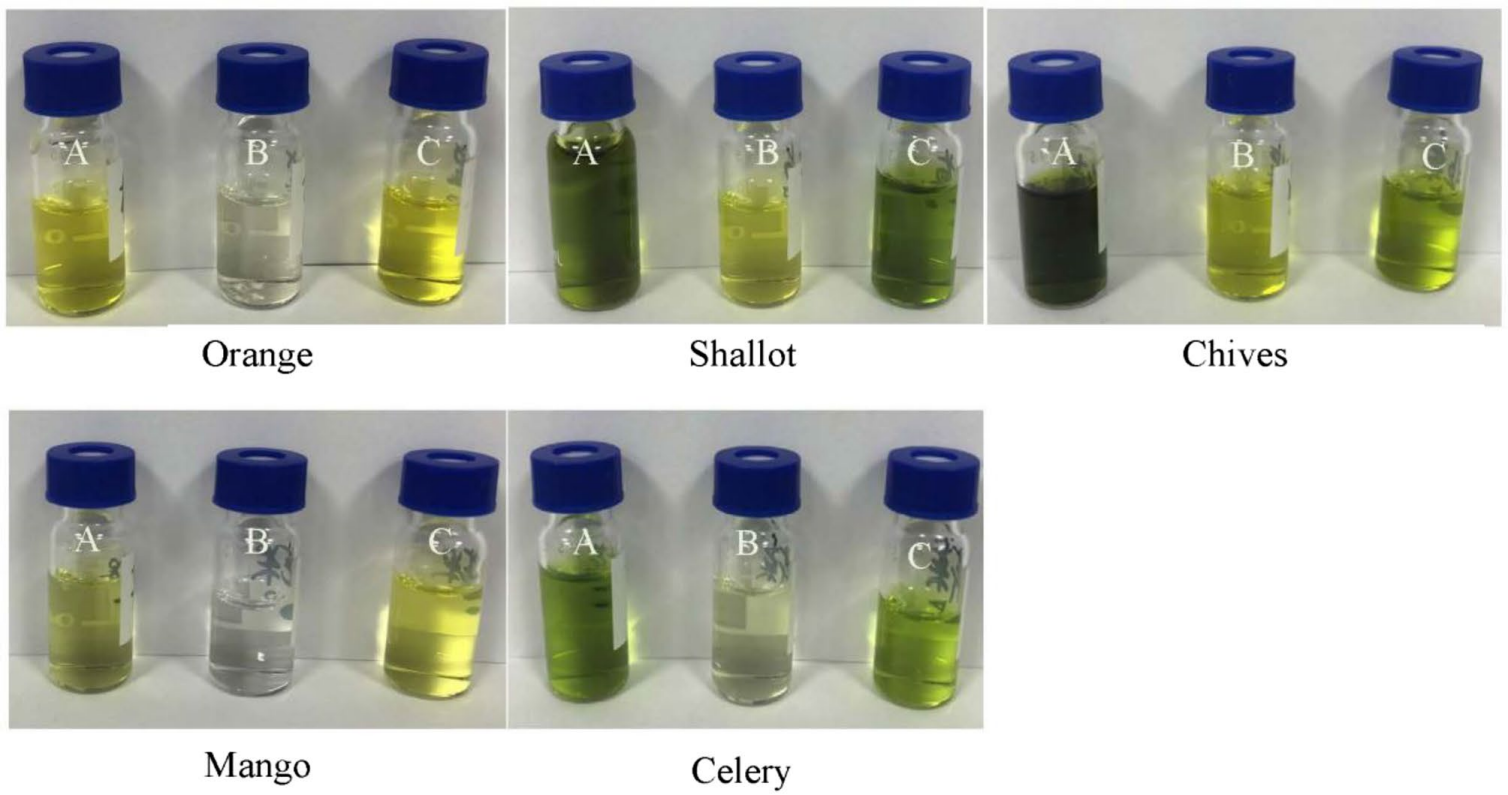
Different purification materials to remove pigment (**A**) without cleanup materials, (**B**) multiwalled carbon nanotubes, (**C**) PSA.

In order to enhance cleanup performance, MWCNTs and PSA were compared. The results are shown in Fig. 6, when PSA were used, except for the low recovery of flonicamid in chives and shallots, tolfenpyrad in avocado, the high recovery of cyantraniliprole in celery, other pesticides were at the acceptable range. On the other hand, for substrates with higher pigment content such as oranges, shallot, chives, mangoes, and celery, which were processed by MWCNT with transparent solution, while the solution with PSA-cleanup had deeper color in Fig. 7. Compared with PSA, the pigment in the sample can be effectively removed by the MWCNT.

### Method validation

#### Linearity

Linearity was studied in the range 0.15–20 ng mL^−1^ for eight amide pesticides by matrix-matched standard calibration in blank extracts of orange, chives, shallot, garlic, mango, onion, avocado, celery, litchi. As shown in Table 3, good linear range was found for all pesticides with *R*^2^ values better than 0.999.

#### Limits of quantification

The described method was tested for simultaneous extraction and determination of eight amide pesticides in nine representative matrices. Table 2 showed the LOQs for the eight pesticides studied in orange, chives, shallot, garlic, mango, onion, avocado, celery, litchi. The LOQs for eight pesticides ranged from 0.03 to 0.8 μg kg^−1^. Tolfenpyrad also had lower LOQs than the other seven pesticides.

**Table 2:** Linear ranges, limits of quantitation (LOQs) and matrix effect of amide pesticides in different matrices.

Matrix	Chlorantraniliprole			Bromoantraniliprole			Flonicamid			Cyantraniliprole			Tolfenpyrad			Flubendiamide			Tetrachlorantraniliprole			Cyclaniliprole		
	Linear ranges (ng mL^−1^)	LOQ (μg kg^−1^)	ME (%)	Linear ranges (ng mL^−1^)	LOQ (μg kg^−1^)	ME (%)	Linear ranges (ng mL^−1^)	LOQ (μg kg^−1^)	ME (%)	Linear ranges (ng mL^−1^)	LOQ (μg kg^−1^)	ME (%)	Linear ranges (ng mL^−1^)	LOQ (μg kg^−1^)	ME (%)	Linear ranges (ng mL^−1^)	LOQ (μg kg^−1^)	ME (%)	Linear ranges (ng mL^−1^)	LOQ (μg kg^−1^)	ME (%)	Linear ranges (ng mL^−1^)	LOQ (μg kg^−1^)	ME (%)
Orange	0.15–20.0	0.15	7.2	0.30–20.0	0.30	5.4	0.80–20.0	0.80	− 8.8	0.20–20.0	0.20	5.5	0.03–20.0	0.03	4.9	0.20–20.0	0.20	− 1.8	0.10–20.0	0.10	3.2	0.50–20.0	0.50	− 6.0
Celery			2.7			6.9			− 9.6			2.3			3.8			9.5			7.6			6.5
Onion			− 0.3			1.0			− 8.1			2.9			4.9			9.0			2.2			6.5
Litchi			6.9			− 0.5			− 2.2			6.2			4.9			8.6			4.3			2.5
Mango			3.0			8.4			1.5			7.2			6.0			6.8			8.6			1.5
Shallot			− 2.7			− 1.0			− 5.1			− 2.9			3.8			5.2			1.1			− 8.0
Chives			9.0			8.9			− 6.6			7.8			− 1.6			8.5			5.4			1.0
Avocado			2.7			8.4			8.8			5.2			− 1.1			8.2			2.2			8.0
Garlic			1.2			3.4			− 8.1			4.5			5.5			8.6			2.2			6.0

#### Recovery and reproducibility

All the recoveries were determined from the analyses of eight amide pesticides in the matrices, orange, chives, shallot, garlic, mango, onion, avocado, celery, litchi by carrying out six consecutive extractions (*n* = 6) of spiked matrices at three concentration levels (LOQs, 5 × LOQs, 10 × LOQs). The values were calculated using matrix-matched calibration standards, as Table 3 shows detailed recovery and relative standard deviation data for eight pesticides analyzed in the nine matrices. The recoveries of eight pesticides were in the range 71.2–120.0% with the relative standard deviations (RSDs) were in the range 3.8–9.4% for all cases.

**Table 3:** Recoveries and relative standard deviations of amide pesticides.

Matrix	Chlorantraniliprole		Bromoantraniliprole		Flonicamid		Cyantraniliprole		Tolfenpyrad		Flubendiamide		Tetrachlorantraniliprole		Cyclaniliprole	
	Added (μg kg^−1^)	Recovery/%( RSD/%)	Added (μg kg^−1^)	Recovery/% (RSD/%)	Added (μg kg^−1^)	Recovery/% (RSD/%)	Added (μg kg^−1^)	Recovery/% (RSD/%)	Added (μg kg^−1^)	Recovery/% (RSD/%)	Added (μg kg^−1^)	Recovery/% (RSD/%)	Added (μg kg^−1^)	Recovery/% (RSD/%)	Added (μg kg^−1^)	Recovery/% (RSD/%)
Orange	0.15	99.9 (6.4)	0.30	98.5 (8.7)	0.80	72.0 (6.4)	0.20	101 (8.2)	0.03	98.9 (6.4)	0.20	99.4 (5.0)	0.10	111 (5.8)	0.50	98.3 (5.2)
	0.75	94.3 (5.6)	1.50	96.7 (7.8)	4.00	75.4 (7.5)	1.00	91.3 (6.4)	0.15	94.6 (5.7)	1.00	101 (5.4)	0.50	106 (6.9)	2.50	95.7 (6.1)
	1.50	96.7 (5.9)	3.00	99.4 (5.6)	8.00	77.9 (4.6)	2.00	95.4 (6.9)	0.30	96.7 (6.0)	2.00	99.7 (4.2)	1.00	117 (4.6)	5.00	97.9 (5.6)
Celery	0.15	106 (7.2)	0.30	103 (7.7)	0.80	78.0 (6.3)	0.20	120 (8.3)	0.03	87.7 (5.7)	0.20	92.5 (5.8)	0.10	110 (6.3)	0.50	94.2 (6.3)
	0.75	101 (7.7)	1.50	100 (5.4)	4.00	79.1 (6.9)	1.00	110 (6.7)	0.15	89.5 (4.9)	1.00	97.3 (6.4)	0.50	112 (3.9)	2.50	99.1 (5.7)
	1.50	103 (6.7)	3.00	105 (4.5)	8.00	88.3 (4.8)	2.00	107 (7.5)	0.30	94.5 (5.2)	2.00	95.8 (4.6)	1.00	107 (4.7)	5.00	96.8 (4.8)
Onion	0.15	73.7 (6.9)	0.30	99.0 (9.1)	0.80	78.1 (7.9)	0.20	76.4 (7.6)	0.03	82.1 (5.3)	0.20	81.9 (7.2)	0.10	90.2 (6.8)	0.50	78.8 (6.9)
	0.75	77.7 (5.3)	1.50	95.7 (8.4)	4.00	88.7 (6.7)	1.00	86.5 (5.6)	0.15	87.3 (5.9)	1.00	83.6 (6.8)	0.50	98.8 (5.0)	2.50	83.5 (5.4)
	1.50	83.5 (6.2)	3.00	99.7 (6.3)	8.00	85.3 (7.5)	2.00	84.7 (5.1)	0.30	89.9 (4.9)	2.00	88.6 (5.4)	1.00	96.9 (5.4)	5.00	87.9 (5.8)
Litchi	0.15	93.8 (5.9)	0.30	91.8 (9.4)	0.80	78.5 (6.7)	0.20	87.6 (7.4)	0.03	92.7 (4.8)	0.20	101 (7.4)	0.10	116 (6.9)	0.50	97.8 (7.4)
	0.75	97.3 (4.6)	1.50	96.4 (7.5)	4.00	77.8 (7.3)	1.00	89.5 (4.9)	0.15	90.8 (4.3)	1.00	100 (5.7)	0.50	112 (4.6)	2.50	96.6 (5.9)
	1.50	95.6 (4.8)	3.00	95.3 (8.8)	8.00	83.1 (5.4)	2.00	90.4 (5.0)	0.30	95.9 (4.0)	2.00	97.8 (8.3)	1.00	117 (5.2)	5.00	98.1 (6.1)
Mango	0.15	92.8 (4.8)	0.30	95.0 (8.4)	0.80	99.0 (6.3)	0.20	101 (7.9)	0.03	96.1 (4.3)	0.20	100 (6.4)	0.10	111 (7.5)	0.50	97.4 (7.3)
	0.75	95.3 (5.1)	1.50	94.2 (4.7)	4.00	94.5 (7.2)	1.00	91.9 (7.7)	0.15	95.8 (6.5)	1.00	108 (5.8)	0.50	115 (6.3)	2.50	95.6 (5.6)
	1.50	98.2 (4.5)	3.00	96.7 (5.2)	8.00	95.6 (6.6)	2.00	96.3 (6.6)	0.30	97.4 (4.8)	2.00	105 (5.6)	1.00	112 (5.9)	5.00	98.9 (6.0)
Shallot	0.15	94.0 (9.1)	0.30	100 (8.8)	0.80	74.1 (4.8)	0.20	91.3 (6.4)	0.03	93.0 (4.9)	0.20	91.2 (5.9)	0.10	114 (7.2)	0.50	89.0 (8.5)
	0.75	97.3 (6.8)	1.50	98.3 (6.1)	4.00	77.4 (6.3)	1.00	94.6 (5.5)	0.15	96.2 (5.3)	1.00	96.0 (5.5)	0.50	115 (5.6)	2.50	93.4 (5.8)
	1.50	98.5 (7.5)	3.00	103 (6.9)	8.00	79.6 (4.0)	2.00	98.4 (5.3)	0.30	95.6 (6.7)	2.00	95.6 (4.5)	1.00	118 (4.8)	5.00	96.5 (6.3)
Chives	0.15	113 (8.4)	0.30	117 (9.2)	0.80	77.5 (5.3)	0.20	108 (6.0)	0.03	82.9 (5.8)	0.20	100 (9.0)	0.10	113 (7.4)	0.50	87.5 (3.8)
	0.75	111 (5.8)	1.50	114 (7.5)	4.00	75.2 (5.9)	1.00	105 (6.7)	0.15	88.6 (4.7)	1.00	98.0 (5.6)	0.50	116 (5.0)	2.50	89.3 (4.3)
	1.50	108 (6.0)	3.00	118 (6.7)	8.00	79.3 (4.7)	2.00	109 (5.5)	0.30	89.7 (5.0)	2.00	95.9 (5.7)	1.00	119 (5.5)	5.00	93.2 (4.5)
Avocado	0.15	106 (5.3)	0.30	111 (6.5)	0.80	73.6 (5.1)	0.20	99.5 (5.9)	0.03	76.5 (6.3)	0.20	103 (8.9)	0.10	112 (6.9)	0.50	97.7 (4.4)
	0.75	110 (5.9)	1.50	115 (6.9)	4.00	78.9 (6.3)	1.00	96.8 (5.6)	0.15	79.7 (5.7)	1.00	109 (5.4)	0.50	105 (4.2)	2.50	95.4 (4.7)
	1.50	108 (6.8)	3.00	113 (5.5)	8.00	79.4 (4.5)	2.00	97.7 (4.6)	0.30	87.9 (4.8)	2.00	105 (6.5)	1.00	107 (5.9)	5.00	98.5 (5.3)
Garlic	0.15	73.5 (5.9)	0.30	75.1 (5.8)	0.80	71.9 (6.8)	0.20	84.9 (5.8)	0.03	71.2 (6.1)	0.20	83.1 (8.6)	0.10	95.7 (5.7)	0.50	81.6 (5.3)
	0.75	78.6 (5.3)	1.50	78.3 (5.6)	4.00	83.4 (7.4)	1.00	89.3 (3.9)	0.15	73.6 (5.6)	1.00	88.8 (6.8)	0.50	97.2 (4.5)	2.50	87.4 (4.2)
	1.50	86.8 (4.5)	3.00	76.7 (4.5)	8.00	84.5 (6.0)	2.00	90.5 (4.9)	0.30	76.5 (4.9)	2.00	89.6 (5.0)	1.00	98.8 (4.3)	5.00	92.8 (5.8)

## Conclusions

In this work, a modified QuEChERS method was developed for the purification by MWCNTs and determination of eight amide pesticides by UHPLC-MS/MS applied in complex matrix of agricultural food. The validation parameters of the method in terms of analytical range, precision, recovery and precision showed that the proposed method meets the requirements for pesticide analysis (average recovery values were in the range 71.2–120.0% with RSDs lower than 10%). MWCNTs have been shown to effectively reduce the matrix effect and remove pigments in the matrix. The method was fast, simple, sensitive and suitable for routine analysis.
